# Awareness levels of breast cancer among female university and medical college students in Sylhet city of Bangladesh

**DOI:** 10.1002/cnr2.1608

**Published:** 2022-02-04

**Authors:** Mst. Farzana Akter, Mohammad Ohid Ullah

**Affiliations:** ^1^ Department of Statistics Shahjalal University of Science and Technology Sylhet Bangladesh

**Keywords:** breast cancer, cancer education, epidemiology, women's cancer

## Abstract

**Background:**

Breast cancer has become a concerning health problem worldwide due to its increasing incidence rate. Women from developing countries are dying off due to the lack of knowledge on breast cancer and its different early detection programs.

**Aims:**

This study explores the level of knowledge about breast cancer risk factors, early warning signs, screening, and therapeutic approaches and their influential determinants among university and medical college students.

**Methods:**

A cross‐sectional study was conducted, including 567 female university‐level students (343 female students from 1 university and 224 female students from 1 medical college). This study used a semi‐structured questionnaire about four aspects (risk factors, early warning signs, screening, and therapeutic approaches) of breast cancer, latent class analysis (LCA), and latent class regression (LCR) for investigation.

**Results:**

The percent of knowing correct answers of risk factors, early warning signs, screening approaches, and therapeutic methods were 86.3%, 69.8%, 70.2%, and 51.2%, respectively for medical students who had a high level of awareness and those for university students were 73.0%, 66.8%, 35.9%, and 24.7%. On the other hand, only 37.95% of medical students had been practicing Breast self‐examination (BSE), while it was 18.37% for university students. The most effective predictors of the high level of awareness were age, advertisements (ad) promoting awareness about breast cancer, programs/campaign related to breast cancer, and personal breast problem history.

**Conclusions:**

Taken together, the awareness level about four aspects of breast cancer is low among university students and is moderately high among medical students. Therefore, relevant health education programs in every educational institute are urgently needed to improve the awareness levels among female students to improve women's health at home and abroad.

## 1 INTRODUCTION

Breast cancer is in number second in the most occurring cancers list among humans and in the first place among women. In 2018, the percentage of breast cancer among newly diagnosed cases in both genders was 12.3%, and in women, it was 25.4% (excluding non‐melanoma skin cancer).^1^ In Belgium, the age‐standardized incidence rate of breast cancer was 113.2, which is the highest per 100 000 women, and this rate was 17.0 in Bangladesh.^2^ The incidence rate of breast cancer has an increasing trend, and it will increase in the future, as some prediction tools suggest.^3^, ^4^, ^5^ In Bangladesh, clinical data warehousing is not rich,^6^ but the information collected by different international organizations depicts that the incident rate is increasing speedily in Bangladesh. Moreover, the mortality rate is increasing because of the absence of breast cancer awareness in women, faulty screening tests, outdated medical treatment, and people not having faith in the medical procedure.^7^, ^8^ In many developing countries, the picture of breast cancer patients depicts that doctors detect most breast cancer cases in the last stage of cancer because patients do not know the early warning signs and screening approaches of breast cancer. An early test of cancer leads to early detection of breast cancer and increases the chance of survival. Simple screening approaches like breast self‐examination and clinical breast examination are helpful to detect breast cancer, and knowledge of these approaches among people can compel authorities to employ early detection programs in a region.^9^

The common warning signs of breast cancer are a lump in the breast or under the armpit, pulling in the breast, pain in the breast/nipple, puckering/dimpling breast skin, bleeding or discharge from the nipple, rash in the nipple or breast skin, redness of breast skin, and changes in the size of the breast due to swelling.^10^, ^11^ The exact reason/factor behind unusual changes in the breast still can not be stated.^12^ Nevertheless, clinical and observational researchers pointed out some like being a woman, early menstruation, diet, late menopause, infertility, family history of cancer, overweight, smoking and alcohol consumption, physically inactive, not breastfeeding, trauma.^13^, ^14^, ^15^, ^16^, ^17^, ^18^, ^19^, ^20^, ^21^, ^22^ To detect any abnormalities in the breast as early as possible, doctors suggest three screening approaches, and those are (a) breast self‐examination (BSE), (b) clinical breast examination (CBE), and (c) mammogram. Along with these, CT scans/sonography and biopsy are also used to confirm the abnormalities to be signs of breast cancer.^23^, ^24^, ^25^, ^26^ Treatment of breast cancer depends on the type and stage of this cancer. Most breast cancer cases require surgical treatment‐ breast‐conserving surgery, mastectomy. Along with these surgeries, surgeons perform other necessary surgeries to remove cancer cells entirely, and doctors prescribe therapies either before or after the surgery.^27^

The knowledge about the risk factors, early warning signs, screening methods and therapeutic methods can be varied respective to a person's educational level, economic status, family history of breast cancer and marital status.^28^ Also, several campaigns/interventions are showing fruitful results in improving the knowledge about breast cancer and encouraging women to practice screening approaches in their lives.^29^, ^30^

Many studies suggested the strong relation of level of awareness with the early detection of breast cancer,^31^, ^32^ and most people had no interest in being conscious about screening approaches for breast cancer.^32^ However, developed countries provided that healthcare experts could explain the need for screening approaches to general people and increase the number of women attending the screening sessions.^33^, ^34^ Several studies^10^, ^11^, ^35^, ^36^, ^37^, ^38^, ^39^, ^40^, ^41^, ^42^, ^43^, ^44^, ^45^ in the world aimed to discover the awareness levels of breast cancer among women. As far as we know, there is no such study conducted in Bangladesh that measured the awareness levels about risk factors, early warning signs, screening approaches, and therapeutic methods among women. Most of the studies conducted in Bangladesh only focused on obtaining the percentages of different factors influencing breast cancer.

This study aims to find out the current awareness level about breast cancer among university‐level female students. Moreover, to identify which factors affect the awareness level of students. As we know, they have more access to various mediums to know the details of this than other sections of the population. Hopefully, this study will help policymakers plan intervention strategies to make women more aware of various aspects of breast cancer.

## 2 METHODOLOGY

We conducted a cross‐sectional study on undergraduate and graduate‐level students in Sylhet city in the northeastern part of Bangladesh. There are six universities and five medical colleges, among which two universities and one medical college are public institutions in this city. Due to limited resources and lack of cooperation, the study population was female students of one university and one medical college. We considered students of five academic years (1st year, 2nd year, 3rd year, 4th year, Master's) of all departments from the university. Similarly, we included Medical College students of five academic years (1st year, 2nd year, 3rd year, 4th year, 5th year) and intern doctors.

There were two groups in the population, and between them, one group had medical knowledge, and the other group had no such knowledge. The group with medical knowledge included female students of medical college, and the group with no such knowledge included the female students of the university. The calculation of sample sizes of the two groups followed the following procedure.

We applied simple random sampling to collect a comparable and representative sample from each group without equal respondents in two groups. To get the required number of samples, we considered the level of significance, *d* = 0.05, Z‐score for 95% confidence interval, *z* = 1.96, and *N =* Population size.

As we had no idea about the proportions aware of breast cancer, we selected *p* = 0.05, and so, *q* = 1 – *p* = .05. When estimating the sample size, the population size for the group having medical knowledge was *N =* 535 and for the other group receiving no medical knowledge was *N =* 3160. For calculating the total number of respondents included in the sample from two assemblies, we used the following equations^46^:

$$n_{0}=\frac{z^{2}\mathrm{pq}}{d^{2}}\;\text{and}\;n=\frac{Nn_{0}}{N+n_{0}}$$

Thus, the sample size for the group having medical knowledge for our study was 224, and the sample size of the other group was 343.

We developed a semi‐structured questionnaire with various information about breast cancer and took different vital questions from the Breast Module of the Cancer Awareness Measure, developed by Cancer Research UK, King's College London, and University College London in 2009. Moreover, the questions of the last five sections were based on the American Cancer Society^27^ and different published articles.^10^, ^11^, ^47^ This questionnaire contained six sections, each one named with its proper heading indicating its topic. These sections had questions about socio‐demographic information, breast cancer risk factors (RF), early warning signs (EWS) of breast cancer, screening approaches (SA) and therapeutic methods (TM) of breast cancer, and barriers to seeking medical help related to breast. The first section contained socio‐demographic variables: age, height, weight, the institution of graduation, marital status, attending any breast cancer‐related educational campaigns or programs, seeing breast cancer‐promoting ads, and personal breast‐related problems. The second section had questions on risk factors, and the third section had questions on early warning signs of breast cancer. The fourth section had questions on screening methods, and the fifth section comprised Likert scale questions on different reasons for not taking medical help. Furthermore, the sixth section included questions for assessing the knowledge about therapeutic methods of breast cancer. All the questions used for the assessment of awareness levels were recorded as “Yes” and “No” options.

We collected data from the halls of female students, which the educational institutions provide. We included students of different academic years randomly from February 2, 2019 to March 15, 2019. Data collectors went room to room and described the objective of the study. A student received a questionnaire if she was interested in participating voluntarily; otherwise was not bothered. We completed the data collection procedure by taking the necessary time allowing flexibility to the respondents.

Data processing steps included data entry, data editing, and necessary adjustment for data analysis. For data processing steps, we used Statistical Package for Social Sciences (SPSS) version 22.0. We edited data on some sections‐ where respondents gave unfitting or meaningless answers and some places to get consistent results among respondents.

We did descriptive analyses and stated mean (±*SD*) for numerical variables and percentage for categorical variables. We considered the structure of the dataset as a latent structure as we selected variables from the sections of risk factors, early warning signs, screening approaches and therapeutic methods, which are categorical variables with two categories‐ “Yes” and “No”. We wanted to find out distinct classes with similar understanding/knowledge among individuals and the chance of participants being in different classes. Furthermore, we analyzed this by applying latent class analysis (LCA) method to each section of the dataset individually to fulfill the aim. LCA was developed for this setting where multiple categorical variables describe one idea and a hidden categorical variable that influences those multiple variables. Participants were assessed based on having adequate or low knowledge about items of each section, and the correct answer for each question was finalized before applying the LCA. Then to measure the extent of effects of factors on the awareness levels about breast cancer, we used latent class regression (LCR), the extended version of LCA. LCR uses the constructed categories by the LCA as the dependent variable for running logistic regression. For factors, obtained coefficients can be converted into odds ratio (OR) to interpret them simply as the participant's chance of being in another category than the reference category. In this study, we used six factors that somehow connected to the awareness level of breast cancer. These factors are the age of respondents, marital status, personal breast problem history, ads promoting awareness about breast cancer, educational program/campaign, and relative with breast cancer. We used the statistical modeling program Mplus version 7.0 to run this part of the analysis.

## 3 RESULTS

### 3.1 Awareness levels of medical students

The mean (±*SD*) age of participants of the medical college was 21.61(1.56) years, and mean (±*SD*) height and weight were 1.58 (±0.05) meters and 53.04 (±9.10) kg, respectively. Among 224 medical students, the majority of them (97.32%) were single, 76.34% were never attending any programs/campaigns related to breast cancer, and a total of 75% of students saw ads that promoted awareness of breast cancer. In addition, 91.96% of students had no family history of breast cancer, and 62.05% did not practice breast self‐examination (Table 1).

**TABLE 1:** Descriptive information about medical students

Variables	Categories		N (%)/Mean (±*SD*)	
Age in years			21.61 (1.56)	
Height in inch			62.10 (2.01)	
Weight in kg			53.04 (9.10)	
BMI	Underweight		41 (18.30)	
	Normal		128 (57.14)	
	Overweight		31 (13.84)	
	Pre‐obese		21 (9.38)	
	Obese T‐1		2 (0.89)	
	Obese T‐2		1 (0.45)	
Marital status	Single		218 (97.32)	
	Married		6 (2.68)	
Attending program/campaign	Yes		53 (23.66)	
	No		171 (76.34)	
Seen awareness related Ads	Yes		168 (75.00)	
	No		56 (25.00)	
Trauma	Yes		25 (11.16)	
	No		199 (88.84)	
Know TM	Yes		156 (69.60)	
	No		68 (30.36)	
Men_BC	Yes		176 (78.57)	
	No		48 (21.43)	
Personal breast problem history	Yes	Breast lump	1 (0.45)	12 (5.36)
		Inflammation	1 (0.45)	
		Itching	1 (0.45)	
		Mastalgia	1 (0.45)	
		Unequal size	1 (0.45)	
		Pain	6 (2.68)	
		Rash in breast skin	1 (0.45)	
	No		212 (94.64)	
Member with breast cancer	Yes	Mother	3 (1.33)	18 (8.04)
		Aunt	9 (4.02)	
		Sister	1 (0.45)	
		Cousin	3 (1.33)	
		Grandmother	2 (0.89)	
	No		206 (91.96)	
Diet	Yes	Balanced Diet	1 (0.45)	26 (11.61)
		Less calorie food	1 (0.45)	
		Not mentioned	12 (5.36)	
		Not specific one	1 (0.45)	
		OMAD	4 (1.79)	
		Vegetarian	7 (3.13)	
	No		198 (88.39)	
Exercise	Yes	Daily	26 (11.61)	119 (53.13)
		Sometimes	78 (34.82)	
		Rarely	17 (7.59)	
	No		105 (46.88)	
Taught to do BSE	Yes	Parents	5 (2.23)	131 (58.48)
		Teacher	90 (40.18)	
		Friend	8 (3.57)	
		Ads	1 (0.45)	
		NGO agent/organization	2 (0.89)	
		Book	4 (1.79)	
		Internet	18 (8.04)	
		Magazine	1 (0.45)	
		TV/Newspaper	2 (0.89)	
	No		93 (41.52)	
Practice BSE	Yes	Weekly	2 (0.89)	85 (37.95)
		Monthly	39 (17.42)	
		Occasionally	29 (12.95)	
		Rarely	15 (6.70)	
	No		139 (62.05)	
Ever done mammogram	Yes		5 (2.23)	
	No	Not old enough	47 (20.98)	219 (97.77)
		Financial constraint	5 (2.23)	
		Not interested	84 (37.50)	
		Others	83 (37.05)	
Total	224 (100.00)			

LCA produced two latent classes for medical students and calculated the percentage of being in a class to give one of two answers to breast cancer risk factors. Table 2 shows the prevalence of the correct answers to the questions about specific breast cancer screening approaches in constructed classes. After observing different percentages in these classes, the classes can be easily labeled based on awareness levels. Here, class‐1 contained 86.3% of participants and included students with relatively high knowledge about breast cancer risk factors, and class‐2 included 13.7% of participants having low knowledge level. Thus, in class‐2, the chances of answering “Yes” to questions about risk factors were relatively low. However, for not breastfeeding, the case was much high; even so, the chance was lower than that for class‐1. Furthermore, in the meantime, for class‐1, the percentage of answering “Yes” to any of the 11 questions was high enough.

**TABLE 2:** Awareness (%) of medical students about RF into latent classes and size of classes

Risk factors (RF)	Class‐1 (high awareness)		Class‐2 (low awareness)	
	Yes	No	Yes	No
Early Menarche	53.3%	46.7%	19.2%	80.8%
Diet	58.0%	42.0%	22.7%	77.3%
Late menopause	73.2%	26.8%	31.0%	69.0%
Family history of breast cancer	93.3%	6.7%	67.5%	32.5%
Infertility	78.1%	21.9%	29.2%	70.8%
Being overweight/obese	86.7%	13.3%	53.4%	46.6%
Smoking	96.2%	3.8%	0.0%	100.0%
Alcohol consumption	94.5%	5.5%	4.4%	95.6%
Not breast feeding	94.3%	5.7%	70.7%	29.3%
Trauma	65.2%	34.8%	29.0%	71.0%
Not being physically active	73.0%	27.0%	48.4%	51.6%
Class size	86.3%		13.7%	

Through applying LCA on the responses of different early warning signs of breast cancer of medical students, we evaluate respondents' knowledge level about these warning signs. We found two classes, among which one class includes those respondents who have high awareness about these, and the other includes respondents with low awareness (Table 3). The class‐1 included most of the respondents, with 69.8%. From the correct answers to the questions about warning signs, we found two classes where class‐1 included respondents with high awareness and class‐2 had individuals with insufficient knowledge. We observed that the chance of answering “Yes” to questions about early warning signs in class‐1 was relatively high. In addition to this, the percentage of answering “Yes” was 100% for “lump or thickening in the breast” in class‐1. Whereas in class‐2, the chance of answering “Yes” was relatively low for each question, but for lump or thickening in the breast, something other than milk in the nipple, and pain in breasts/armpit, the chances were high.

**TABLE 3:** Awareness (%) of medical students about EWS into latent classes and size of classes

Early warning signs (EWS)	Class‐1 (high awareness)		Class‐2 (low awareness)	
	Yes	No	Yes	No
Pulling in of nipple	77.8%	22.2%	19.9%	80.1%
Pain in breasts/armpit	82.6%	17.4%	60.4%	39.6%
Puckering/dimpling breast skin	92.5%	7.5%	28.6%	71.4%
Bleeding or discharge something other than milk in nipple	94.7%	5.3%	62.1%	37.9%
Lump or thickening in breast	100.0%	0.0%	77.8%	22.2%
Nipple rash	57.3%	42.7%	30.2%	69.8%
Redness of breast skin	59.2%	40.8%	40.7%	59.3%
Lump or thickening under armpit	90.1%	9.9%	55.0%	45.0%
Change in size of breast	84.5%	15.5%	54.6%	45.4%
Class size	69.8%		30.2%	

The percentage of having accurate knowledge about screening approaches and practicing those approaches to detect breast cancer as early as possible among medical students was portrayed (Table 4). Applying LCA to answers given by medical students provides two classes representing awareness levels. Class‐1 having 70.2% of respondents, consisted of individuals with high awareness, and class‐2 had 29.8% of respondents with low awareness. In class‐2, the chance of answering “Yes” to “practice BSE” was meager, and the percentage of answering “Yes” for this question in class‐1 was about half. The chances of answering “Yes” in class‐2 were relatively low for other questions than in class‐1, but the values were not that small.

**TABLE 4:** Awareness (%) of medical students about SA into latent classes and size of classes

Screening approaches (SA)	Class‐1 (high awareness)		Class‐2 (low awareness)	
	Yes	No	Yes	No
Know about breast self‐examination (BSE)	100.0%	0.0%	53.6%	46.4%
Practice BSE	51.0%	49.0%	7.3%	92.7%
Clinical breast‐examination (CBE)	86.7%	13.3%	41.5%	58.5%
Mammogram	93.7%	6.3%	43.0%	57.0%
Biopsy	93.1%	6.9%	57.9%	42.1%
CT scan/sonography	76.3%	23.7%	61.5%	38.5%
Class size	70.2%		29.8%	

From the data of medical students, after applying LCA, we discovered two distinct latent classes and the percentage of correct answers to questions about therapeutic methods given by respondents in each latent group (Table 5). Class‐1 represented individuals having high awareness, and class‐2 represented those with low awareness about breast cancer. The size of class‐1 and class‐2 was 51.2% and 48.8%, respectively. The chance of answering “yes” to “Surgery or removal of the whole or part breast” was 100% for class‐1, while for class‐2, it was 18.6%. Although the chances of answering “Yes” to “Alternative medicine” were low for both classes, the opportunity was meager for class‐1.

**TABLE 5:** Awareness (%) of medical students about TM into latent classes and size of classes

Therapeutic methods (TM)	Class‐1 (high awareness)		Class‐2 (low awareness)	
	Yes	No	Yes	No
Surgery or removal of whole or part of breast	100.0%	0.0%	18.6%	81.4%
Chemical or radiotherapy	98.3%	1.7%	11.3%	88.7%
Depends on disease stage	98.3%	1.7%	11.3%	88.7%
Alternative medicine	1.7%	98.3%	4.6%	95.4%
Class size	51.2%		48.8%	

The classes of LCA were used in the logistic regression of LCR and the second class was the reference category. After applying LCR, we discovered that for risk factors of breast cancer, the age of respondents had a significant positive effect on being a respondent in the high awareness group (OR = 1.63; 95% CI = [1.21, 2.21]). Furthermore, having a family history of breast cancer also positively affected being in the high awareness group, but the impact was not significant (OR = 3.16; 95% CI = [0.30, 33.33]). Also, we could see that other factors had no positive or significant effect on being in class‐1. Determinants having a negative impact, but not notable were personal breast problem history (OR = 0.34), being married (OR = 0.35), educational program/campaign related to breast cancer (OR = 0.52), and ads that promote awareness about breast cancer (OR = 0.63). For early warning signs of breast cancer, the age of respondents significantly affected the respondents to be in high awareness class (OR = 1.57; 95% CI = [1.16, 2.13]). Having seen ads that promote breast cancer awareness positively affected being in high awareness class, but the impact was not significant (OR = 2.05; 95% CI = [0.89, 4.70]). OR portrayed that Respondents having relatives with breast cancer tended to be in the high awareness class, and the association had not been found significant (OR = 1.43; 95% CI = [0.39, 5.25]). Moreover, being married (OR = 0.18), personal breast problem history (OR = 0.68), and program/campaign related to breast cancer (OR = 0.94) had a negative effect, although the impacts were not significant (Table 6).

**TABLE 6:** Odds ratio (OR) and 95% confidence interval of OR for the effects of different factors on awareness level about different aspects of breast cancer of medical students

Independent variables	Different aspects of breast cancer			
	Risk factors OR (95% CI)	Early warning signs OR (95% CI)	Screening approaches OR (95% CI)	Therapeutic methods OR (95% CI)
Age	1.63^a^ (1.21, 2.21)	1.57^a^ (1.16, 2.13)	2.66^a^ (1.64, 4.31)	1.88^a^ (1.46, 2.42)
Marital status^b^ (married)	0.35 (0.05, 3.53)	0.18 (0.02, 1.42)	0.02 (0.00, 0.25)	0.30 (0.07, 1.38)
Program campaign	0.52 (0.20, 1.32)	0.94 (0.35, 2.50)	1.86 (0.59, 5.88)	1.64 (0.76, 3.53)
Ads	0.63 (0.22, 1.79)	2.05 (0.89, 4.70)	1.80 (0.68, 4.76)	1.62 (0.80, 3.28)
Personal breast problem history	0.34 (0.08, 1.48)	0.68 (0.17, 2.72)	2.38 (0.21, 26.81)	0.79 (0.16, 3.80)
Member with cancer	3.16 (0.30, 33.33)	1.43 (0.39, 5.25)	0.35 (0.06, 1.95)	0.79 (0.25, 2.45)

*Note*: Class‐2 (low level) is reference class.

^a^ *p*‐value <.05.

^b^ Reference category is single.

Same as earlier aspects, the age of respondents had a positive and highly significant effect on respondents being in high awareness class (OR = 2.66; 95% CI = [1.64, 4.31]) in case of screening approaches of breast cancer. Along with this, factors having positive effect but not significant, in order of importance, were personal breast problem history (OR = 2.38; 95% CI = [0.21, 26.81]), program/campaign related to breast cancer (OR = 1.86; 95% CI = [0.59, 5.88]) and ads that promote breast cancer awareness (OR = 1.80; 95% CI = [0.68, 4.76]). It was also evident that being married had a significantly negative impact on an individual in high awareness class. Having a family member/relative with breast cancer had a negative impact but was not significant. As we can see, like other parts, the age of respondents had a significantly positive effect on individuals being in high awareness class (OR = 1.88; 95% CI = [1.46, 2.42]) for therapeutic methods of breast cancer. On the other hand, programs/campaigns related to breast cancer (OR = 1.64) and ads promoting awareness of breast cancer (OR = 1.62) positively but not significantly impacted individuals in high awareness class. Moreover, being married (OR = 0.30), personal breast history problem (OR = 0.79), having a relative with breast cancer (OR = 0.79) had a negative but not significant effect on individuals being in class‐1 (Table 6).

### 3.2 Awareness levels of university students

The mean (±*SD*) age of university female student participants was 22.59 (±1.55) years, and the mean (±*SD*) height and weight of them were 1.56 (±0.06) meters and 51.24 (±8.39) kg. Among 343 female university students, 93.88% were single, and 88.92% of students never attended any breast cancer‐related campaign or programs, but 63.56% had seen ads promoting breast cancer awareness. Around 13% of students had a family history of breast cancer, but only 18.37% practiced breast self‐examination (BSE) (Table 7).

**TABLE 7:** Descriptive information about university students

Variables	Categories		N (%)/Mean (*SD*)	
Age in years	—		22.59 (1.55)	
Height in inch	—		61.40 (2.30)	
Weight in kg	—		51.24 (8.39)	
Marital status	Single		322 (93.88)	
	Married		21 (6.12)	
BMI status	Underweight		76 (22.16)	
	Normal		185 (53.94)	
	Overweight		44 (12.83)	
	Pre‐obese		33 (9.62)	
	Obese T‐1		5 (1.46)	
Attend program/campaign	Yes		38 (11.08)	
	No		305 (88.92)	
Ads	Yes		218 (63.56)	
	No		125 (36.44)	
Member with breast cancer	No		299 (87.17)	
	Yes		44 (12.83)	
		Mother	6 (1.75)	
		Aunt	21 (6.12)	
		Sister	1 (0.29)	
		Cousin	8 (2.33)	
		Others	6 (1.75)	
		Grandmother	6 (1.75)	
Personal breast problem	Yes	Pain	17 (4.96)	27 (7.87)
		Rash in breast skin	5 (1.46)	
		Mastitis	1 (0.29)	
		Soreness	1 (0.29)	
		Discharge something from nipple	1 (0.29)	
		Dimpling in Breast skin	1 (0.29)	
		Breast tumor	1 (0.29)	
	No		316 (92.13)	
Smoking	Yes		6 (1.75)	
	No		337 (98.25)	
Alcohol	Yes		4 (1.17)	
	No		339 (98.83)	
Diet	Yes		24 (7.00)	
	No		319(93.00)	
Exercise	Yes	Daily	21 (6.12)	114 (33.24)
		Sometimes	70 (20.41)	
		Rarely	23 (6.71)	
	No		229 (66.76)	
Trauma	Yes		99 (28.86)	
	No		244 (71.14)	
Men_BC	Yes		148 (43.15)	
	No		195 (56.85)	
Practice BSE	Yes	Monthly	15 (4.37)	63 (18.37)
		Occasionally	31 (9.04)	
		Rarely	17 (4.96)	
	No		280 (81.63)	
Taught to do BSE	Yes	Parents	11 (3.21)	106 (30.90)
		Sister	2 (0.58)	
		Cousin	1 (0.29)	
		Aunt	1 (0.29)	
		Teacher	13 (3.79)	
		Doctor	17 (4.96)	
		Nurse	1 (0.29)	
		Friend	21 (6.12)	
		Internet	34 (9.91)	
		Newspaper	3 (0.87)	
		TV	1 (0.29)	
		Health related program	1 (0.29)	
	No		237 (69.10)	
Ever done Mam	Yes		1 (0.29)	
	No	Not old enough	9 (2.62)	342 (99.71)
		Do not know it	283 (82.51)	
		Not interested	30 (8.75)	
		Financial constraint	5 (1.46)	
		Didn't face any problem	2 (0.58)	
		Didn't find abnormalities	1 (0.29)	
		Didn't think about it	2 (0.58)	
		Not needed	10 (2.92)	
Know TM	Yes		117 (34.11)	
	No		226 (65.89)	
Total	343 (100.00)			

Table 8 shows the percentage of the correct answer to questions about different risk factors given by university students in terms of two latent classes. Class‐2 included individuals with high knowledge about risk factors defined as high awareness class and class‐1 with less knowledgeable individuals. Here, class‐2 contained 73% of study participants, whereas class‐1 had 27%. In class‐1, the percentage of answering “Yes” to each question was lower than in class‐2. Although the percentages of answering “Yes” to questions were higher than class‐1 for some questions, the chances were not high enough such as for Early Menarche, the chance of answering “Yes” was only 33.8% (Table 8).

**TABLE 8:** Awareness (%) of university students about RF into latent classes and size of classes

Risk factors (RF)	Class‐1 (Low awareness)		Class‐2 (High awareness)	
	Yes	Yes	Yes	No
Early Menarche	12.3%	33.8%	33.8%	87.7%
Diet	7.0%	31.8%	31.8%	93.0%
Late menopause	23.5%	51.2%	51.2%	76.5%
Family history of breast cancer	35.3%	76.4%	76.4%	64.7%
Infertility	16.0%	65.2%	65.2%	84.0%
Being overweight/obese	29.7%	60.5%	60.5%	70.3%
Smoking	6.9%	91.0%	91.0%	93.1%
Alcohol consumption	0.0%	88.3%	88.3%	100.0%
Not breast feeding	45.3%	86.3%	86.3%	54.7%
Trauma	17.2%	30.8%	30.8%	82.8%
Not being physically active	26.6%	50.5%	50.5%	73.4%
Class size	27.0%		73.0%	

The LCA assessed the awareness level of university students about early warning signs of breast cancer which provided two latent classes. Regarding the percentage of correct answers to early warning signs in two classes, we could see that class‐1 included individuals with low awareness about warning signs of breast cancer with 33.2% of the study population, and class‐2 contained high aware individuals (66.8%). In class‐1, the probability of answering “Yes” for any of the nine questions was very low, while in class‐2, the probabilities were high enough. For instance, the chance of correct answer for “something other than milk in nipple” was 81.8% in class‐2 while 11% in class‐1 (Table 9).

**TABLE 9:** Awareness (%) of university students about EWS into latent classes and size of classes

Early warning signs (EWS)	Class‐1 (low awareness)		Class‐2 (high awareness)	
	Yes	No	Yes	No
Pulling in of nipple	7.4%	92.6%	40.9%	59.1%
Pain in breasts/armpit	22.3%	77.7%	81.5%	18.5%
Puckering/dimpling breast skin	7.8%	92.2%	69.9%	30.1%
Bleeding or discharge something other than milk in nipple	11.0%	89.0%	81.8%	18.2%
Lump or thickening in breast	4.3%	95.7%	68.6%	31.4%
Nipple rash	7.0%	93.0%	48.1%	51.9%
Redness of breast skin	9.3%	90.7%	51.7%	48.3%
Lump or thickening under armpit	1.3%	98.7%	56.5%	43.5%
Change in size of breast	11.2%	88.8%	69.1%	30.9%
Class size	33.2%		66.8%	

Table 10 shows the percentages of correct answers to the questions about screening approaches in terms of the latent class, and from this prevalence, we could understand that class‐1 contained students with low awareness level and class‐2 consisted of individuals with low awareness level. From the class size, we found that only 35.9% of individuals had a high level of knowledge, and 64.1% had a low level of knowledge about screening approaches for breast cancer. The probability of practicing BSE was 0% in class‐1, while the chance was 51.1% in class‐2. However, the chance of answering “yes” for “CT scan/sonography” was only 35% in class‐2, and in class‐1, the chance was lower than that (Table 10).

**TABLE 10:** Awareness (%) of university students about SA into latent classes and size of classes

Screening approaches (SA)	Class‐1 (low awareness)		Class‐2 (high awareness)	
	Yes	No	Yes	No
Know about breast self‐examination (BSE)	15.6%	84.4%	94.7%	5.3%
Practice BSE	0.0%	100.0%	51.1%	48.9%
Clinical breast‐examination (CBE)	8.4%	91.6%	62.1%	37.9%
Mammogram	2.2%	97.8%	41.6%	58.4%
Biopsy	36.7%	63.3%	61.2%	38.8%
CT scan/sonography	17.2%	82.8%	35.0%	65.0%
Class size	64.1%		35.9%	

The LCA identified distinct classes from study participants of the university regarding the prevalence of correct answers to therapeutic methods. From the percentage of the correct answers in two latent classes, we found that class‐1 contained most of the participants (75.3%) who had a low level of knowledge about therapeutic methods, while class‐2 included individuals with a high level of knowledge (24.7%). For Surgery/removal of whole or part of the breast, the correct answers were 100% in class‐2, while 8.6% in class‐1 (Table 11).

**TABLE 11:** Awareness (%) of university students about TM into latent classes and size of classes

Therapeutic methods (TM)	Class‐1 (low awareness)		Class‐2 (high awareness)	
	Yes	No	Yes	No
Surgery or removal of whole or part of breast	8.6%	91.4%	100.0%	0.0%
Chemical or radiotherapy	2.8%	97.2%	92.9%	7.1%
Depends on disease stage	0.4%	99.6%	97.9%	2.1%
Alternative medicine	0.8%	99.2%	1.2%	98.8%
Class size	75.3%		24.7%	

LCR indicated the impacts of six factors on individuals of the university being in high awareness class. In LCR, latent classes were dependent variables, and class‐1 (low awareness) was the reference class like logistic regression. The obtained coefficients of six factors were easy to interpret as OR of belonging in other classes rather than reference one. The coefficients associated with factors for the aspect “risk factors” of breast cancer showed that being married (OR = 1.82; 95% CI = [0.51, 6.44]), personal breast problem history (OR = 1.77; 95% CI = [0.57, 5.53]), ads promoting awareness about breast cancer (OR = 1.36; 95% CI = [0.76, 2.45]), relative with breast cancer (OR = 1.14; 95% CI = [0.46, 2.83]) and age (OR = 1.05; 95% CI = [0.86, 1.30]) had positive but not significant impact on individuals being in high awareness group rather than reference one while educational program/campaign (OR = 0.90; 95% CI = [0.36, 2.21]) had negative but insignificant effect. All six factors had an insignificant impact on differentiating class‐2 from class‐1. From the results, for the early warning signs, it was clear that ads promoting awareness about breast cancer had a significant positive impact on participants being in high awareness class than reference class (OR = 5.85; 95% CI = [3.20, 10.67]). Though educational program/campaign had more positive impact but the impact was not significant (OR = 10.14; 95% CI = [0.06, 1705.14]). Other factors had not any significant effect on distinguishing the latent classes. The factors with significant positive effect on the level of awareness about screening approaches of breast cancer were personal breast problem history (OR = 6.33; 95% CI = [2.06, 19.46]), promotional ads of breast cancer awareness (OR = 4.31; 95% CI = [2.24, 8.31]), educational program/campaign (OR = 3.38; 95% CI = [1.31, 8.76]) and age (OR = 1.20; 95% CI = [1.01, 1.44]). From the results for therapeutic methods, educational program/campaign had positive significant impact on differentiating two classes (OR = 2.49; 95% CI = [1.26, 4.94]) while being married (OR = 2.17; 95% CI = [0.89, 5.29]), ads promoting awareness (OR = 1.61; 95% CI = [0.93, 2.81]) and relatives with Breast Cancer (OR = 1.79; 95% CI = [0.90, 3.57]) had positive effects which were not much significant (Table 12).

**TABLE 12:** Odds ratio (OR) and 95% confidence interval of OR for the effects of different factors on awareness level about different aspects of breast cancer of university students

Independent variables	Different aspects of breast cancer			
	Risk factors OR (95% CI)	Early warning signs OR (95% CI)	Screening approaches OR (95% CI)	Therapeutic methods OR (95% CI)
Age	1.05 (0.86, 1.30)	1.06 (0.86, 1.31)	1.20^a^ (1.01, 1.44)	1.05 (0.90, 1.23)
Marital status^b^ (married)	1.82 (0.51, 6.44)	0.90 (0.32, 2.49)	1.43 (0.48, 4.25)	2.17 (0.89, 5.29)
Program campaign	0.90 (0.36, 2.21)	10.14 (0.06, 1705.14)	3.38^a^ (1.31, 8.76)	2.49^a^ (1.26, 4.94)
Ads	1.36 (0.76, 2.45)	5.85^a^ (3.20, 10.67)	4.31^a^ (2.24, 8.31)	1.61 (0.93, 2.81)
Personal breast problem history	1.77 (0.57, 5.53)	2.27 (0.72, 7.21)	6.33^a^ (2.06, 19.46)	1.79 (0.79, 4.07)
Member with cancer	1.14 (0.46, 2.83)	1.01 (0.37, 2.75)	1.50 (0.66, 3.43)	1.79 (0.90, 3.57)

*Note*: Class‐1 is reference class.

^a^ *p*‐value <.05.

^b^ Reference category is single.

## 4 DISCUSSION

Breast cancer awareness is paramount to diagnose it early and is the most common cancer among women (excluding skin cancer). Being aware of this cancer can reduce the chance of developing breast cancer. Many organizations worldwide are focusing more on the awareness part of breast cancer. They are organizing various campaigns, online tutorials, booklets to teach people of all ages about the importance of early detection and factors that might influence the development of breast cancer. Early detection of breast cancer helps specialists destroy the cancer cell as early as possible, and the treatment of cancer becomes less costly with a higher possibility of recovery. This investigation explored the level of awareness about the risk factors, early warning signs, screening modalities, and therapeutic approaches taken by medics of breast cancer among university and medical students. It also helped to know different factors which could influence their curiosity to be familiar with other aspects of this cancer.

The current study gives information on the awareness levels about different parts of breast cancer among university and medical students. The knowledge about breast cancer risk factors among university and medical students is high. However, a relatively small number of university students identified trauma, diet, and early menarche as risk factors of breast cancer. In contrast, the least number of medical students did the same for early menarche and diet. Furthermore, a cross‐sectional study on Iranian adult women provided low awareness about breast cancer risk factors.^11^ Another cross‐sectional research on university students of UAE displayed relatively low knowledge about different risk factors before the awareness program, which changed at the end of the study.^35^ Finally, another survey of medical and non‐medical program university students in Angola revealed the same results, depicting the low level of awareness.^36^ Thus, our findings on risk factors are inconsistent with some studies.^11^, ^35^, ^36^, ^37^, ^38^ Our analysis also revealed that the awareness level about early warning signs of breast cancer was high among university and medical students but not high enough among medical students. It is presumed that medical students will have proficient knowledge about the different diseases, and from that assumption, the awareness level is compared. On the other hand, nipple rash and redness of breast skin had a lower percentage of the correct answer to be recognized as an early warning sign by medical students and the same for pulling in of nipple, redness of breast skin, and lump or lump thickening under armpit by university students. Some earlier studies provided lower levels of knowledge about early warning signs than our research.^11^, ^36^, ^37^, ^38^, ^39^ That could be due to the education level^48^ and the availability of sources of breast cancer information for the study participants. Our research discovered adequate knowledge about screening approaches of breast cancer among medical students and insufficient knowledge among university students. Although having high knowledge about screenings, only about half of medical students had been practicing BSE. On the other hand, practicing BSE was least commonly done by university students because of insufficient knowledge. Other than that, the knowledge about mammogram and CT scan/sonography is much low among them. Several studies regarding this topic showed almost similar insufficient knowledge for university students about screening approaches among participants.^11^, ^39^, ^42^ Surprisingly, participants of the university who had high knowledge about screening methods had inadequate knowledge about mammogram and CT scan/sonography. Among medical students who knew the procedure of BSE‐ most of them learned about this process from their teacher/professor, which is quite normal for medical students. This source does not exist for university students, and most of the university students acquired knowledge of this procedure from the internet and mixed sources. Those sources taught them about this process, and they are also actively doing their part. However, we found that different educational programs or campaigns must be held more in various areas or institutions to familiarize women with BSE better.

Our study aimed to discover the awareness about widely‐known curative methods of breast cancer, and the result displayed that awareness level is very low for university students. Surprisingly, the level is also low for medical students. Those university and medical students who had high knowledge about curative procedures gave a high rate of correct answers for each option. A study in India among women of a rural district found low knowledge about therapeutic methods^43^ also among the Iranian adult women.^11^ The literature review revealed few published papers about the knowledge of therapeutic procedures to make a proper comparison. Our findings might be the result of the therapeutic methods being complicated and not known to general people. Also, the therapeutic methods are available for medical students to be informed about after a specific time in their degree period, which can justify this result for medical students. From the overall findings of the level of awareness about four sections of breast cancer, on average, we found high awareness among students compared to other published studies on different groups of populations.

The levels of awareness among participants are varied because of their personal information, and necessary steps can change most of the reasons. For medical students, one common influential factor was the respondent's age, and it is evident that the final year students had better knowledge about the aspects of breast cancer than the first‐year students. Alternatively, for university students, age did not effectively influence the awareness levels, which is usual as they will not get any extra course regarding this topic at any level of their education.

We found a significant but negative association between marital status and awareness about screening approaches among medical students. A study on Jordanian nurses showed no significant association between marital status and performing BSE,^49^ and another study on Saudi adult women showed an insignificant effect of marital status on the knowledge of breast cancer.^45^ On the other hand, a significant association existed between awareness level of screening approaches and marital status, a family member with breast cancer, educational qualification among women in Riyadh.^41^ A study showed a strong association in Australian women between knowledge and practice of screening approach and factors like marital status, personal history of the breast problem, and age. However, it was found that women over 65 years were not interested in doing BSE, and women of the reproductive age group were concerned about examining their breasts.^50^ Our findings might be the consequences of having a low number of married students and different age groups. At the same time, other articles argued in favor of influential association by indicating that married people are usually more worried about their health condition because they have more chance of getting affected by the different refractory disease.

In university students, we found a positive and significant association between age, attending program/campaign, ads promoting awareness about breast cancer, personal breast problem history, and awareness level about screening approaches. Personal breast problem history is an essential factor for awareness about screening approaches because it is evident that after feeling any pain or seeing any unusual changes in the breast, women tend to see doctors. Furthermore, from there, women got to find out different screening methods. It also indicates that students are concerned about their health symptoms and always consult doctors if they find any unusual changes. Moreover, a significant association existed between attending programs/campaigns and awareness levels about therapeutic methods for university students. University students showed that ads promoting awareness about breast cancer could help them get accurate knowledge about breast cancer early warning signs. However, we could not find any significant factors among university students for the knowledge of breast cancer risk factors. In Bangladesh, there are a few ads promoting awareness about any part of breast cancer, and the number of such ads must be increased covering every aspect of breast cancer. Along with this, in each educational institution, programs/campaigns that give details about breast cancer should be organized every year.

As awareness about breast cancer is necessary among women, especially aged 15–49 years, the main strong point of this study is that we want to know the extent of knowledge about this among students in Bangladesh who have more access to materials of the necessary information and this type of study was already done in many other countries but not any significant research in Bangladesh. Besides, the statistical technique used in the current study provides fair and trustworthy results about the levels of awareness and effective factors that vary the awareness level about breast cancer.

The limitation of this study is that only two educational institutes of one city of Bangladesh were used for participants to investigate the awareness levels. This study can be extended by considering more undergraduate and graduate students or more women aged 15–49 years from every district in Bangladesh. This study is conducted using simple random sampling, and further study can be run by using other random sampling techniques and more samples. Cluster sampling procedure may also be used to investigate the breast cancer patients in different divisions in Bangladesh. Also, to get an in‐depth understanding of the factors that influence the awareness among women, mixed‐method research approaches are possible to employ.

## 5 CONCLUSION

The current study revealed a moderately low awareness level about the screening approaches and therapeutic methods among university students and relatively high awareness among medical students. However, the awareness level concerning breast cancer risk factors was moderate among medical and university students. The results exposed the fact that female students having personal breast problem history, attending programs related to breast cancer, seeing informative ads on breast cancer tended to have a high awareness of breast cancer. Educational programs about breast cancer in every educational institute, events at the community level to raise awareness among women, and voluntarily learning about breast cancer from online events, web pages, and tutorials using technology could be practical steps to take.

## CONFLICT OF INTEREST

The authors declare no conflicts of interest.

## AUTHOR CONTRIBUTION

All authors had full access to the dataset used in this study and took full responsibility for the analysis, the accuracy, and the representation of the data. Mst. Farzana Akter: conceptualization, data curation, formal analysis, investigation, methodology, validation, visualization, and writing‐original draft. Mohammad Ohid Ullah: conceptualization, methodology, supervision, validation, writing‐review & editing.

## ETHICS STATEMENT

The study was approved by the ethical/research (Thesis) committee 2018 (Session 2016–2017) [Regi/161(council)/1/2233] of the Department of Statistics department, Shahjalal University of Science and Technology, Sylhet, Bangladesh. The objective of this study was clarified verbally to the students, and they signed a written consent form that was included on the top of the questionnaire. They had been assured that their identity would not be linked with the research materials, and they would not be identified or identifiable in any report.

## Data Availability

The data that support the findings of this study are available on request from the corresponding author. The data are not publicly available due to privacy or ethical restrictions.

## References

[1] Worldwide cancer data. World Cancer Research Fund, 2018. Available from: https://www.wcrf.org/dietandcancer/cancer-trends/worldwide-cancer-data.

[2] Global Cancer Observatory, 2019. Available from: http://gco.iarc.fr/.

[3] Poirier AE , Ruan Y , Walter SD , et al. The future burden of cancer in Canada: long‐term cancer incidence projections 2013–2042. Cancer Epidemiol. 2019;59:199‐207. 30831552 10.1016/j.canep.2019.02.011

[4] Zaheer S , Shah N , Maqbool SA , Soomro NM . Estimates of past and future time trends in age‐specific breast cancer incidence among women in Karachi, Pakistan: 2004–2025. BMC Public Health. 2019;19(1):1001. 31345204 10.1186/s12889-019-7330-z PMC6659231

[5] Study forecasts new breast cancer cases by 2030 ‐ National Cancer Institute, 2015. Available from: https://www.cancer.gov/news-events/cancer-currents-blog/2015/breast-forecast.

[6] Akter MF , Islam T , Trisha KF , Ullah MO . An overview of data mining in medical informatics: Bangladesh perspective. Asian J Med Biol Res. 2019;5(4):258‐264.

[7] Shulman LN , Willett W , Sievers A , Knaul FM . Breast cancer in developing countries: opportunities for improved survival. J Oncol. 2010;2010:e595167. 10.1155/2010/595167 PMC3021855 21253541

[8] WHO supports early detection and control of cervical and breast cancer in Bangladesh, 2021. Available from: https://www.who.int/bangladesh/news/detail/10-11-2020-who-supports-early-detection-and-control-of-cervical-and-breast-cancer-in-bangladesh.

[9] Elmore JG , Armstrong K , Lehman CD , Fletcher SW . Screening for breast cancer. Jama J Am Med Assoc. 2005;293(10):1245‐1256. 10.1001/jama.293.10.1245 PMC3149836 15755947

[10] Madubogwu CI , Egwuonwu AO , Madubogwu NU , Njelita IA . Breast cancer screening practices amongst female tertiary health worker in Nnewi. J Cancer Res Ther. 2017;13(2):268‐275. 28643746 10.4103/0973-1482.188433

[11] Tazhibi M , Feizi A . Awareness levels about breast cancer risk factors, early warning signs, and screening and therapeutic approaches among Iranian adult women: A large population based study using latent class analysis. BioMed Res Int. 2014;2014. doi:10.1155/2014/306352 PMC4180890 25295257

[12] Risk factors. National Breast Cancer Foundation, 2021. Available from: https://www.nationalbreastcancer.org/breast-cancer-risk-factors/.

[13] Khatib OMN , Modjtabai A . Guidelines for the early detection and screening of breast cancer. World Health Organization, Regional Office for the Eastern Mediterranean, 2006. Available from: https://apps.who.int/iris/handle/10665/119805.

[14] Collaborative Group on Hormonal Factors in Breast Cancer . Familial breast cancer: collaborative reanalysis of individual data from 52 epidemiological studies including 58,209 women with breast cancer and 101,986 women without the disease. Lancet Lond Engl. 2001;358(9291):1389‐1399. 10.1016/S0140-6736(01)06524-2 11705483

[15] Cetin I , Cozzi V , Antonazzo P . Infertility as a cancer risk factor ‐ a review. Placenta. 2008;29:169‐177. 18790330 10.1016/j.placenta.2008.08.007

[16] Picon‐Ruiz M , Morata‐Tarifa C , Valle‐Goffin JJ , Friedman ER , Slingerland JM . Obesity and adverse breast cancer risk and outcome: mechanistic insights and strategies for intervention. CA Cancer J Clin. 2017;67(5):378‐397. 28763097 10.3322/caac.21405 PMC5591063

[17] Jones ME , Schoemaker MJ , Wright LB , Ashworth A , Swerdlow AJ . Smoking and risk of breast cancer in the generations study cohort. Breast Cancer Res BCR. 2017;19(1):118. 29162146 10.1186/s13058-017-0908-4 PMC5698948

[18] Kabat GC , Kim M , Phipps AI , et al. Smoking and alcohol consumption in relation to risk of triple‐negative breast cancer in a cohort of postmenopausal women. Cancer Causes Control CCC. 2011;22(5):775‐783. 21360045 10.1007/s10552-011-9750-7 PMC3100347

[19] Stuebe A . The risks of not breastfeeding for mothers and infants. Rev Obstet Gynecol. 2009;2(4):222‐231. 20111658 PMC2812877

[20] Rigby JE , Morris JA , Lavelle J , Stewart M , Gatrell AC . Can physical trauma cause breast cancer? Eur J Cancer Prev off J Eur Cancer Prev Organ ECP. 2002;11(3):307‐311. 10.1097/00008469-200206000-00014 12131664

[21] McTiernan A . Behavioral risk factors in breast cancer: can risk be modified? Oncologist. 2003;8(4):326‐334. 12897329 10.1634/theoncologist.8-4-326

[22] Kotepui M . Diet and risk of breast cancer. Contemp Oncol. 2016;20(1):13‐19. 10.5114/wo.2014.40560 PMC4829739 27095934

[23] Breast self‐exam. National Breast Cancer Foundation, 2021. Available from: https://www.nationalbreastcancer.org/breast-self-exam/.

[24] Clinical breast exam. National Breast Cancer Foundation, 2021. Available from: https://www.nationalbreastcancer.org/clinical-breast-exam/.

[25] Mammogram. National Breast Cancer Foundation, 2021. Available from: https://www.nationalbreastcancer.org/mammogram/.

[26] Breast cancer diagnosis. National Breast Cancer Foundation, 2021. Available from: https://www.nationalbreastcancer.org/breast-cancer-diagnosis/.

[27] Breast cancer | Breast cancer information & overview. American Cancer Society, 2019. Available from: https://www.cancer.org/cancer/breast-cancer.html.

[28] Alam NE , Islam MS , Ullah H , et al. Evaluation of knowledge, awareness and attitudes towards breast cancer risk factors and early detection among females in Bangladesh: A hospital based cross‐sectional study. PLOS One. 2021;16(9):e0257271. 34516589 10.1371/journal.pone.0257271 PMC8437277

[29] Anastasi N , Lusher J . The impact of breast cancer awareness interventions on breast screening uptake among women in the United Kingdom: A systematic review. J Health Psychol. 2019;24(1):113‐124. 28810435 10.1177/1359105317697812

[30] Glynn RW , Kelly JC , Coffey N , Sweeney KJ , Kerin MJ . The effect of breast cancer awareness month on internet search activity ‐ a comparison with awareness campaigns for lung and prostate cancer. BMC Cancer. 2011;11(1):442. 21993136 10.1186/1471-2407-11-442 PMC3210140

[31] Cruz‐Castillo AB , Hernández‐Valero MA , Hovick SR , Campuzano‐González ME , Karam‐Calderón MA , Bustamante‐Montes LP . A study on the knowledge, perception and use of breast cancer screening methods and quality of care among women from Central Mexico. J Cancer Educ off J Am Assoc Cancer Educ. 2015;30(3):453‐459. 10.1007/s13187-014-0722-y PMC4362850 25182506

[32] Dey S , Mishra A , Govil J , Dhillon PK . Breast cancer awareness at the community level among women in Delhi, India. Asian Pac J Cancer Prev. 2015;16(13):5243‐5251. 26225660 10.7314/apjcp.2015.16.13.5243

[33] Lurie N , Margolis KL , McGovern PG , Mink PJ , Slater JS . Why do patients of female physicians have higher rates of breast and cervical cancer screening? J Gen Intern Med. 1997;12(1):34‐43. 9034944 10.1046/j.1525-1497.1997.12102.x PMC1497051

[34] Bekker H , Morrison L , Marteau TM . Breast screening: GPs' beliefs, attitudes and practices. Fam Pract. 1999;16(1):60‐65. 10321398 10.1093/fampra/16.1.60

[35] Abduelkarem AR , Saif FK , Saif S , Alshoaiby TA . Evaluation of breast cancer awareness among female university students in University of Sharjah, UAE. Adv Breast Cancer Res. 2015;9(4):9‐21.

[36] Sambanje MN , Mafuvadze B . Breast cancer knowledge and awareness among university students in Angola. Pan Afr Med J. 2012;11(70). https://www.ncbi.nlm.nih.gov/pmc/articles/PMC3361208/ PMC3361208 22655104

[37] Liu L‐Y , Wang F , Yu L‐X , et al. Breast cancer awareness among women in eastern China: a cross‐sectional study. BMC Public Health. 2014;14(1004). doi:10.1186/1471-2458-14-1004 PMC4180963 25257142

[38] Kan'an A . Evaluation of breast cancer (BC) awareness among female university students in Zarqa University. Jordan Eur J Breast Health. 2018;14(4):199‐204. 30288493 10.5152/ejbh.2018.4048 PMC6170026

[39] Salaudeen AG , Akande T , Musa O . Knowledge and attitudes to breast cancer and breast self examination among female undergraduates in a state in Nigeria. Eur J Soc Sci. 2009;1:7.

[40] Linsell L , Burgess CC , Ramirez AJ . Breast cancer awareness among older women. Br J Cancer. 2008;99(8):1221‐1225. 18813307 10.1038/sj.bjc.6604668 PMC2570528

[41] Alam AA . Knowledge of breast cancer and its risk and protective factors among women in Riyadh. Ann Saudi Med. 2006;26(4):272‐277. 16883082 10.5144/0256-4947.2006.272 PMC6074496

[42] Montazeri A , Vahdaninia M , Harirchi I , et al. Breast cancer in Iran: need for greater women awareness of warning signs and effective screening methods. Asia Pac Fam Med. 2008;7(1):6. 19099595 10.1186/1447-056X-7-6 PMC2628874

[43] Gangane N , Ng N , Sebastian MS . Women's knowledge, attitudes, and practices about breast cancer in a Rural District of Central India. Asian Pac J Cancer Prev. 2015;16(16):6863‐6870. 26514458 10.7314/apjcp.2015.16.16.6863

[44] Donnelly TT , Khater A‐HA , Al‐Bader SB , et al. Factors that influence awareness of breast cancer screening among Arab women in Qatar: results from a cross sectional survey. Asian Pac J Cancer Prev. 2014;15(23):10157‐10164. 25556441 10.7314/apjcp.2014.15.23.10157

[45] Amin TT , Al AM , Al AM . Breast cancer knowledge, risk factors and screening among adult Saudi women in a primary health care setting. Asian Pac J Cancer Prev. 2009;10(1):133‐138. 19469641

[46] Islam MN . An Introduction to Sampling Methods: Theory and Applications. Book World; 2005:494.

[47] Wachira J , Busakhala A , Chite F , et al. Refining a questionnaire to assess breast cancer knowledge and barriers to screening in Kenya: psychometric assessment of the BCAM. BMC Health Serv Res. 2017;17(1):110. 28158984 10.1186/s12913-017-2058-x PMC5291974

[48] Malik R , Vera N , Dayal C , et al. Factors associated with breast cancer awareness and breast self‐examination in Fiji and Kashmir India – a cross‐sectional study. BMC Cancer. 2020;20(1):1078. 33167928 10.1186/s12885-020-07583-w PMC7654031

[49] Hadayat A . Breast self‐examination and risk factors of breast cancer: awareness of jordanian nurses. Health Sci J. 2013;7(3):303‐314.

[50] Yelland MJ , Rice DE , Ward AE , Bain C , Siskind V , Schofieid F . A profile of Australian women practicing breast self‐examination. Asia Pac J Public Health. 1991;5(4):307‐312. 1844220 10.1177/101053959100500409

